# Polypharmacy among people living with type 2 diabetes mellitus in rural communes in Vietnam

**DOI:** 10.1371/journal.pone.0249849

**Published:** 2021-04-08

**Authors:** Dieu Huyen Thi Bui, Bai Xuan Nguyen, Dat Cong Truong, Dan Wolf Meyrowitsch, Jens Søndergaard, Tine Gammeltoft, Ib Christian Bygbjerg, Nielsen Jannie

**Affiliations:** 1 Faculty of Public Health, Thai Binh University of Medicine and Pharmacy, Thai Binh, Vietnam; 2 Department of Embryology, Thai Binh University of Medicine and Pharmacy, Thai Binh, Vietnam; 3 Department of Public Health, Global Health Section, University of Copenhagen, Copenhagen K, Denmark; 4 Department of Public Health, The Research Unit for General Practice, University of Southern Denmark, Odense C, Denmark; 5 Department of Anthropology, University of Copenhagen, Copenhagen K, Denmark; 6 Hubert Department of Global Health, Emory Global Diabetes Research Center, Rollins School of Global Health, Emory University, Atlanta, GA, United States of America; University of South Australia, AUSTRALIA

## Abstract

**Objectives:**

People with diabetes are at high risk of polypharmacy owing to complex treatment of diabetes and comorbidities. Polypharmacy is associated with increased risk of adverse reactions and decreased compliance. Therefore, the objectives of this study were to assess polypharmacy in people with type 2 diabetes (T2D) and associated diabetes-related factors in rural areas in Vietnam.

**Method:**

People with T2D (n = 806) who had received treatment for diabetes at a district hospital were invited to participate in a questionnaire-based cross-sectional survey. Polypharmacy was defined as ≥5 types of medicine and assessed as a) prescription medicine and non-prescription/over the counter (OTC) medicine and b) prescription medicine and non-prescription/OTC, herbal and traditional medicine, and dietary supplement. Multiple logistic regression was used to investigate the association between polypharmacy and diabetes specific factors: duration, comorbidities and diabetes-related distress.

**Results:**

Of the people with T2D, 7.8% had a medicine use corresponding to polypharmacy (prescription medicine and non-prescription/OTC), and 40.8% when herbal and traditional medicine, and dietary supplement were included. Mean number of medicine intake (all types of medicines and supplements) were 3.8±1.5. The odd ratios (ORs) of polypharmacy (medicine and supplements) increased with diabetes duration (<1–5 years OR = 1.66; 95%CI: 1.09–2.53 and >5 years OR = 1.74; 95%CI: 1.14–2.64 as compared to ≤1-year duration of diabetes), number of comorbidities (1–2 comorbidities: OR = 2.0; 95%CI: 1.18–3.42; ≥3 comorbidities: OR = 2.63;95%CI: 1.50–4.61 as compared to no comorbidities), and suffering from diabetes-related distress (OR = 1.49; 95%CI: 1.11–2.01) as compared to those without distress.

**Conclusions:**

In rural northern Vietnam, persons with longer duration of T2D, higher number of comorbidities and diabetes-related stress have higher odds of having a medicine use corresponding to polypharmacy. A high proportion of people with T2D supplement their prescription, non-prescription/OTC medicine with herbal and traditional medicine and dietary supplements.

## Introduction

The prevalence of diabetes among adults in Vietnam has increased from 2.7% in 2002 [1] to 6% in 2017 [2]. To prevent complications and comorbidities such as hypertension, heart disease and arthritis [2,3], people with diabetes must daily adhere to glucose-lowering medicine, monitor and test their blood glucose, address cardiovascular disease risk factors, including blood pressure [4] and lipid management [5,6], and often significantly change their diet and physical activity level [7]. Consequently, people living with diabetes often have to use multiple medicines for their diabetes, comorbidities and complications [8]. Concomitant use of multiple medicines can be harmful even when each drug is prescribed according to clinical guidelines because each additional medicine increases the risk of adverse reactions and decreased compliance [9].

The use of multiple medications is defined as “polypharmacy” [10]. There is no consensus on the definition of polypharmacy: polypharmacy has been defined both excluding [11] and including none-Western medications such as herbal and traditional medicine and dietary supplements [12]. Studies defining polypharmacy as only prescribed/OTC medicine have reported that polypharmacy is associated with adverse drug events, drug-drug interaction, hospitalization, cost [13], mortality, and morbidity [14]. Previous studies also showed that people with diabetes more often have medication use corresponding to polypharmacy than people without diabetes [11,15]. Further, polypharmacy reduces adherence to the medicine and decreases the quality of life in people with diabetes [14].

The majority of studies on polypharmacy in people with diabetes have been conducted in high-income countries, and very few have focused on middle-income countries such as Vietnam. A study from Vietnam showed that the rate of polypharmacy among hospitalized patients in general aged 60 years and older was 59.2% [16]. The proportion of people with diabetes with polypharmacy is not known. Therefore, a cross-sectional study was conducted to assess the proportion of adult Vietnamese age ≥40 year with type 2 diabetes (T2D) who have a medicine intake corresponding to polypharmacy and identify factors associated with polypharmacy.

## Materials and methods

### Study population and setting

A cross-sectional survey was conducted from December 2018 to February 2019 in Thai Binh Province, a province in North-eastern Vietnam. The total population is 1.86 million people, GDP per capita 1.650 USD, and more than 90% of the population live in rural areas [17]. Thai Binh Province is subdivided into seven districts and one provincial city and has a total of 9 townlets, 10 wards, and 267 communes.

The state health care system in Vietnam is organized as a four-tiered pyramid. At the top of the pyramid is the Ministry of Health, which subsumes provincial, district and commune health authorities. Thai Binh Province has 12 district hospitals and 2 private hospitals, which are equivalent to district hospitals. People with diabetes can receive treatment at any of these hospitals and they can change the hospital anytime they want.

### Sampling method

Among the seven districts of Thai Binh Province, we selected two districts, Quynh Phu District in the north and Vu Thu District in the south. In each district, the two communes with the highest number of people with T2D were selected. Of these communes, we selected two neighboring commues as these were convenient for data collection. Thus, out of 68 towns/communes in two districts, 8 were selected.

People with T2D residing in the 8 selected communes who had received treatment for their diabetes at the 3 district hospitals in Quynh Phu District and Vu Thu District were invited to take part in the study. Information on name, address, and phone number for these people were based on hospital records and provided by the district hospitals. In Vietnam, hospitals diagnoses diabetes using the WHO criteria. From the 8 communes, 963 people with T2D were invited to participate in the study; 37 respondents refused to participate in the study (3.8%), while 78 respondents could not be found at the reported address or had relocated (8.1%). In Vietnam, age of onset of T2D has been shown to be in the fourth to fifth decade of life [18], while type 1 diabetes is generally diagnosed at younger ages [2]. Our target population was people with T2D, thus, to minimize the potential of misclassification we excluded those with diabetes diagnosed before 40 years of age and those who did not remember their age when diagnosed (n = 42, 4.4%). These criteria resulted in a cohort of 806 people with T2D (respondence rate: 83.7%).

### Ethical approval

The study was approved by the Medical Ethics Committee of Thai Binh University of Medicine and Pharmacy, Vietnam, (decision 11/2018, 23rd November 2018). Written informed consent was obtained from each participant before data collection. Participants could withdraw at any time during the interview. The participants were interviewed in their homes or another place selected by them. The completed questionnaires were managed and stored securely at Thai Binh University of Medicine and Pharmacy, Vietnam.

### Data collection

Data were collected using a structured and pilot-tested questionnaire. We trained 16 village health workers from 8 health stations in the 8 selected communes as interviewers at a 2-day workshop followed by field-based training and counseling to administer the questionnaires. The interviews were as arranged through the staff of the commune health station. To ensure the questions were culturally appropriate and could be understood by the study participants, the questionnaire was pilot tested first among village health workers and second among people admitted to the hospital for diabetes.

### Study variables

#### Outcome

The number of medicines was reported by the person with T2D during the interview, who was encouraged to report prescription medicines, OTC medicines they purchased, herbal and traditional medicine, and dietary supplements, and to show all the medicines they were taking daily.

*Definition of polypharmacy*. There is no general consensus for the definition of polypharmacy, but daily intake of ≥5 medicines is the most commonly used to defined polypharmacy [11,12,19]. However, the definition of what constitutes medicines varies by either excluding or including non-western medicines such as herbal and traditional medicine and dietary supplements [11,12]. In the present study, we created two variables for polypharmacy: a) one only including prescribed medicine and non-prescription/over the counter medicine (OTC); and b) a variable including prescribed medicine and non-prescription/OTC medicine, herbal and traditional medicine, and dietary supplements [12]. Polypharmacy was categorized as: no (0), yes (1). For modeling purposes, we used the second definition as herbal and traditional medicine, and dietary supplements are commonly used in Vietnam.

#### Exposure

Our main exposures of interest were variables related to the participant’s T2D: duration of diabetes, physical health, number of comorbidities, and diabetes-related distress. All this information was self-reported. Comorbidities were coded as 0, 1–2 comorbidities, 3 or more comorbidities) [20]; and duration of diabetes as ≤1 year, >1–5 years, >5 years) [21]. Diabetes-related distress (no, yes) [22] was defined according to the Problem Areas in Diabetes 5 (PAID-5) scale [23].

#### Covariates

Covariates were factors known to be associated with polypharmacy and were categorized as follows: Age (categorized as 40–54 years, 55–69 years, ≥70 years) [15], sex (man, woman) [11], education (primary school and below, middle school, college and above) [24]; economic status (poor/near-poor, medium, wealthy) [25]; occupation (unemployed, farmer, small trade/worker/government employee/private company, retired), marital status (married/living together, single/widowed/divorced) [26], and self-reported physical health (excellent/good, fair, poor/very poor) [27].

### Statistical analysis

Data were double entered using Epidata version 3.1 (The Epidata Association), cleaned and exported to SPSS (IBM Statistical Package for Social Science software) version 22 for analysis. Statistics of continuous variables were described using mean and standard deviation (SD), and categorical variables were summarized by calculating the number and frequency distribution. Bivariate logistic regression analysis was used to calculate the odd ratios (ORs) for the association between polypharmacy and independent variables and only those independent variables satisfied p-value ≤0.1. Next, multiple logistic regressions were adjusted for all independent variable satisfied p-value ≤0.1 in the bivariate logistic regressions. Adjusted odds ratios (AOR) with a 95% confidence interval (95% CI) and p-values <0.05 were used to determine if a risk factor was statistically significantly associated with polypharmacy.

## Results

Out of 806 people living with T2D, 425 (52.7%) were women. The majority of the respondents were in the age group 55–69 years (58.1%). Mean age of the participants was 65.2±9.0 years. Mean duration of diabetes was 5.9 years. One-third (33.3%) of respondents suffered from more than three comorbidities. Half of the respondents (50.0%) presented with diabetes-related distress.

### Polypharmacy

Mean number of medicines was 3.8 (1.5) (including prescription/OTC medicine, herbal and traditional medicine, and dietary supplements), ranging from 0 to 9 medicines per individual (distribution of prescription/OTC medicines:1: 6.6%; 2: 15.1%; 3: 16.6%; 4: 52.7%; ≥5: 7.8%). Table 1 shows that 7.8% of participants had medicine intake corresponding to polypharmacy (using the definition including only prescribed medicine and OTC medicine), while 40.8% had polypharmacy according to the definition also including herbal and traditional medicine, and dietary supplements.

**Table 1:** Medicine use among patients with Type 2 diabetes in Thai Binh, Vietnam.

	No n (%)	Yes n (%)
Only Prescription/OTC^1^	301 (37.3)	505 (62.7)
Prescription/OTC^1^ + Herbal and Traditional Medicine	3 (0.3)	803 (99.7)
Prescription/OTC^1^ + Dietary Supplements	6 (0.7)	800 (99.3)
Prescription/OTC^1^ + Herbal, Traditional Medicine, Dietary Supplements	3 (0.3)	803 (99.7)
Only Herbal and Traditional Medicine	800 (99.3)	6 (0.7)
Only Herbal and Traditional Medicine and Dietary Supplements	800 (99.3)	6 (0.7)
Only Dietary Supplements	806 (100.0)	0 (0.0)
Polypharmacy only including prescription/OTC^1^ medicine	743 (92.2)	63 (7.8)
Polypharmacy including all types of medication (prescribed and non-prescription/OTC^1^ medicine, herbal and traditional medicine, dietary supplement)	477 (59.2)	329 (40.8)

1: OTC: Over the counter.

Table 2 shows the characteristics of people with T2D with and without polypharmacy (including herbal and traditional medicine and dietary supplements). Sex, marital status, occupation, education level, economic status and self-report of physical health did not differ between people with and without polypharmacy. Polypharmacy was associated with duration of diabetes, increasing number of comorbidities, and diabetes-related distress.

**Table 2:** Proportion of people living with T2D with polypharmacy according to demographic, economic and health characteristics.

Characteristic	Total (n = 806)	Polypharmacy^1^		P-value
		No (n = 477)	Yes (n = 329)	
Sex				
Men	381	228 (59.8)	153 (40.2)	0.718
Women	425	249 (58.6)	176 (41.4)	
Age group (years)				
40–54	92	63 (68.5)	29 (31.5)	0.156
55–69	468	271 (57.9)	197 (42.1)	
≥70	246	143 (58.1)	103 (41.9)	
Educational level				
Primary and below	180	117 (65.0)	63 (35.0)	0.131
Middle school	500	292 (58.4)	208 (41.6)	
College and above	126	68 (54.0)	58 (46.0)	
Marital status				
Married/living together	593	354 (59.7)	239 (40.3)	0.619
Single/widowed/divorced	213	123 (57.7)	90 (42.3)	
Occupation (n = 798)^2^				
Unemployed and stays at home	140	82 (58.6)	58 (41.4)	0.622
Farmer	296	180 (60.8)	116 (39.2)	
Small trade/worker/private company/government employee	104	65 (62.5)	39 (37.5)	
Retired	258	145 (56.2)	113 (43.8)	
Economic status (self-reported)				
Poor/near poor	95	52(54.7)	43 (45.3)	0.641
Medium	638	381 (59.7)	257 (40.3)	
Wealthy	73	44 (60.3)	29 (39.7)	
Duration of diabetes (year)				
≤1 year	152	106 (69.7)	46 (30.3)	0.024
>1–5 years	310	180 (58.1)	130 (41.9)	
> 5 years	344	191 (55.5)	153 (44.5)	
Number of comorbidities				
0	92	70 (76.1)	22 (23.9)	<0.001
1–2	446	267 (59.9)	179 (40.1)	
≥3	268	140 (52.2)	128 (47.8)	
Diabetes-related distress^3^				
No	403	256 (63.5)	147 (36.5)	0.012
Yes	403	221 (54.8)	182 (45.2)	
Self-reported level of physical health				
Excellent/Good	105	62 (59.0)	43 (41.0)	0.99
Fair	399	237 (59.4)	162 (40.6)	
Poor/very poor	302	178 (58.9)	124 (41.1)	

^1^ Polypharmacy: No: 0 to 4 medicines in use, Yes: ≥5 medicines in use.

^2^: 8 people had missing information about occupation.

^3^: Diabetes-related distress: Yes: Paid-5 score ≥ 8; No: Paid-5 score <8.

### Factors associated with polypharmacy

The analysis of logistic regression indicated that the factors associated with polypharmacy were duration of diabetes above 1 year, presence of more than 1 comorbidity, and having diabaetes-related distress (Table 3). Compared to those without comorbidities, the ORs of polypharmacy in people with 1–2 comorbidities and ≥3 comorbidities were 2.01 and 2.63 respectively. Polypharmacy was associated with duration of diabetes: compared to those who had diabetes for ≤ 1 year, those with diabetes for 1–5 years and >5 years had higher ORs of polypharmacy was 1.66 and 1.74, respectively. People with diabetes-related distress had higher OR of polypharmacy as compared to those without diabetes-related distress (OR = 1.49).

**Table 3:** Factors associated with polypharmacy.

Characteristic	Number of individuals with polypharmacy^1^ (%)	Results of bivariate analysis Crude odds ratio (95% CI)	Results of multivariate analysis Adjusted odds ratio* (95% CI)
Duration of diabetes (years)			
≤1 year	46 (30.3)	1	1
>1–5 years	130 (41.9)	1.66 (1.01–2.51)	1.66 (1.09–2.53)
> 5 years	153 (44.5)	1.84 (1.23–2.77)	1.74 (1.14–2.64)
Comorbidities			
0	22 (23.9)	1	1
1–2	179 (40.1)	2.13 (1.27–3.57)	2.01 (1.18–3.42)
3 or more	128 (47.8)	2.90 (1.70–4.97)	2.63 (1.50–4.61)
Diabetes-related distress^2^			
No	147 (36.5)	1	1
Yes	182 (45.2)	1.43 (1.08–1.90)	1.49 (1.11–2.01)

^1^ Polypharmacy: No: 0 to 4 medicines in use, Yes: ≥5 medicines in use.

^2^: Distress: Yes: Paid-5 score ≥ 8; No: Paid-5 score <8.

CI: Confident interval.

*Adjusted for duration of diabetes, commorbidities, diabetes-related distress.

## Discussion

This study shows that among people with T2D in rural Vietnam, 7.8% has polypharmacy when defined as ≥5 prescribed/OTC medicines, and 40.8% when also including herbal, traditional medicine and dietary supplements in the polypharmacy definition. Polypharmacy was associated with longer duration of diabetes, increasing number of comorbidities and diabetes related to distress. There was no association between polypharmacy and demographic or socio-economic factors.

Studies from other countries have found the proportion of polypharmacy among people with diabetes range from 48% to 85% [11,15,21,28–30]. However, as definitions of polypharmacy vary, these data are not directly comparable. Thus, these studies defined polypharmacy as either prescription or OTC medicine [11], or only including prescribed medicine. When we defined polypharmacy including only prescribed medicines and OTC, only 7.8% had polypharmacy. This discrepancy may be partly explained by our study population: we included people with T2D who received treatment at a district hospital; in the Thai Binh, people with diabetes with more severe complications usually receive treatment at the provincial or national hospitals [31]. Moreover, most people with diabetes in Vietnam use health insurance to cover treatment and medicine for their disease and the insurance only covers the cost for a monthly check-up in district hospitals up to VND 223,500 (approximately 10 USD), including blood testing, medical examination fee, and medicine [32]. Thus, doctors may prescribe only the most important medicine/s to keep the cost within the limit.

When defining polypharmacy by including herbal and traditional medicine, the proportion of people with diabetes with polypharmacy was 40.8%, which suggests that people with substitute, supplement, or rely on non-pharmaceutical treatment for their diabetes. A study from Vietnam reported that reasons for this include people believing that a combination of conventional and herbal and traditional medicine will improve the effectiveness of their diabetes treatment [33]. A study conducted in the United States among Vietnamese immigrants showed that people with T2D even stop taking prescribed medicine when using traditional medicine because of worries of side effects of prescribed medicines [34]. Further, in Vietnam, using herbal and traditional medicine is considered a therapy with minimal or no side effect and entails lower financial costs [35,36]. This may be explained by traditional and herbal medicine having a long and honored history in Vietnam: for thousands of years, Vietnamese people have treated diseases with herbs and plants, which were gathered from gardens and forests. Thus, using traditional and herbal medicine is still an important component of national efforts to promote public health in urban and rural populations in Vietnam [37]. Nowadays, using traditional and herbal medicine are widely used beside Western medicine and traditional and herbal medicine are especially used when Western medicine is considered ineffective or too expensive [37].

In our study, longer duration of diabetes increased the ORs of polypharmacy. In studies conducted in China, Brazil and Italy among people with T2D showed that polypharmacy was associated with duration of T2D of ≥5 years [15,21,38]. Other studies have also showed an association between diabetes duration and diabetes- related complications and commorbidities [39,40]. Longer duration is associated with increasing risk of diabetes complications and comorbidities [41], which may require additional medicine [42]. The requirement of additional medicine may also explain our results showing that the ORs of polypharmacy were associated with higher number of comorbidities, which is in line with recently published studies [15,21,43–45]. People with T2D are at high risk of arterial hypertension, cardiovascular disorder, hyperlipidemia, ect.,—all conditions, which require medicine: antihypertensive, antihyperlipidemic, antiplatelets medicines are often co-prescribed along with antidiabetic medicine [46].

We found that the OR of polypharmacy was higher among those with diabetes-related distress than those without diabetes-related distress. The results of studies showing that polypharmacy is associated with psychological distress in general [11,22]. People with less well-controlled diabetes may have higher distress due to the difficulties managing their disease and therefore try out different medicines in an effort to better control their disease [36]. On the other hand, polypharmacy may increase the risk of hospitalizations, morbidity and financial cost [47], which may increase the emotional and financial stress for people with diabetes, especially those living in rural Vietnam areas where the average monthly income is around 100 US$ [36,48].

### Limitations and strengths

To our knowledge, this study is the first to report about polypharmacy in people with T2D in Vietnam. We collected data in rural and semi-urban areas and had a response-rate of 84%.

This study also has some limitations; selection bias may have occurred since we only included patients who had received treatment for T2D at district hospitals, which may have excluded 1) people who control their diabetes with diet and physical activity only, and are not in contact with a district hospital; and 2) people who receive diabetes treatment at regional hospital or provincial hospitals, which typically treat more severe cases of diabetes. These people would likely have lower or higher OR of polypharmacy, respectively, and we might have overestimated or underestimated the proportion of people with T2D with polypharmacy. Furthermore, non-responders constituted around 13.5% of the sample, which may have influenced the results: we did not systematically collect data about them, but they mostly reported being unavailable for interview due to being at work, and may thus have been younger and in better health, and have had a lower medicine use than the people included in the study. This study examined the association of polypharmacy including prescription/OTC medicines, herbal, traditional medicine and dietary supplement, the results could be different if the study was limited to only people using prescription/OTC medicines. Our study was conducted in Vietnam and the results cannot be generalized to other populations. Lastly, we do not know the types of medicine which people with T2D were taking and we cannot assess if they were over or undertreated.

## Conclusions

Less than 10% of the study population reported a medicine intake corresponding to polypharmacy defined as prescription and non-prescription/OTC medicine, while the proportion increased to 41% when including herbal and traditional medicine and dietary supplements. Polypharmacy was more common among those persons with longer duration of T2D, higher number of comorbidities and diabetes-related distress. The study findings suggest that a high proportion of people with T2D supplement their treatment with herbal and traditional medicine and dietary supplements. Further research should focus on whether people with T2D in Vietnam are undertreated or overtreated, and if those with polypharmacy experience harmful effects.

## Supporting information

S1 Table Frequency of other disease among peole with T2D. (PDF)

S1 File Questionnaire of study. (PDF)

S2 File Data of study. (XLSX)
