# Supplementary material for: Polypharmacy among people living with type 2 diabetes mellitus in rural communes in Vietnam
Source: PLoS One. 2021 Apr 8;16(4):e0249849. doi: 10.1371/journal.pone.0249849 (PMC8031303; doi:10.1371/journal.pone.0249849)
Supplement: S1 Table — (PDF) [file pone.0249849.s001.pdf]

S1. Frequency of other disease among peole with T2D

Characteristic	Total (n=806)	Polypharmacy[1]	
		No (n=477)	Yes (n=329)
Number of comorbidities			
0	92 (11.4)	70 (14.7)	22 (6.7)
1	210 (26.1)	136 (28.5)	74 (22.5)
2	236 (29.3)	131 (27.5)	105 (31.9)
3	161 (20.0)	94 (19.7)	67 (20.4)
4	86 (10.7)	39 (8.2)	47 (14.3)
5	18 (2.2)	6 (1.3)	12 (3.6)
6	3 (0.4)	1 (0.2)	2 (0.6)
Type of comorbidity			
Hypertension*	458 (56.8)	240 (50.3)	218 (66.3)
Epilepsy	4 (0.5)	3 (0.6)	1 (0.3)
Depression	4 (0.5)	1 (0.2)	3 (0.9)
Tuberculosis	9 (1.1)	6 (1.3)	3 (0.9)
Liver and kindney disease	255 (31.6)	141 (29.6)	114 (34.7)
Bone and joint problem	438 (54.3)	248 (52.0)	190 (57.8)
Cardio-vascular disease*	230 (28.5)	120 (25.2)	110 (33.4)
Other chronic disease*	219 (27.2)	113 (23.7)	106 (32.2)

Asterisks (*) represent significant differences in polypharmacy from χ^2 test, $^*p < 0{,}05$
