# Supplementary material for: Polypharmacy among people living with type 2 diabetes mellitus in rural communes in Vietnam
Source: PLoS One. 2021 Apr 8;16(4):e0249849. doi: 10.1371/journal.pone.0249849 (PMC8031303; doi:10.1371/journal.pone.0249849)
Supplement: S1 File — (PDF) [file pone.0249849.s002.pdf]

Informal support for people living with diabetes

QUESTIONNAIRE 1

‘

Name:
Village:
Commune:
Identification number (if known):
STUDY INFORMANT ID No:_______

DATE OF INTERVIEW (dd/mm/yyyy): …..…/……../……….

000. RECORD THE TIME THE INTERVIEW BEGINS	Hour [ ][ ] (24 h)<br>Minutes [ ][ ]
01. Name of interviewer<br><br>02. Name of supervisor	

STUDY INFORMANT ID No: __________

SECTION 1
THE RESPONDENT AND HIS/HER HOUSEHOLD

QUESTIONS & FILTERS		CODING CATEGORIES	NOTES
If you don't mind, I would like to start by asking you a little about yourself and the household that you live in. Interviewer circles the selected option(s).			
101	Gender of respondent	Male = 0; Female = 1	
102	Date and year of birth?<br><br>'Unknow' date is marked as 99<br>'Unknow' month is marked as 99<br>'Unknow' year is marked as 9998<br>'Refused/no answer' is marked as 9999	DATE OF BIRTH: ___/_____/_____ (DD/MM/YYYY)<br>LUNAR DATE OF BIRTH: ___/_____/_____ (DD/MM/YYYY)	
102a:	If date of birth unknown:<br>How old are you?	AGE (YEARS): ___; DON'T KNOW = 888; REFUSED/NO ANSWER = 999	
103	Where did you grow up?<br><br>PROBE: Before age 18 where did you live longest?	THIS COMMUNE = 1<br>ANOTHER COMMUNE = 2<br>ANOTHER DISTRICT = 3<br>ANOTHER PROVINCE OR CITY = 4<br>DON'T KNOW/DON'T REMEMBER = 8<br>REFUSED/NO ANSWER = 9	
104	What is the highest level of education that you finished?<br><br>MARK HIGHEST LEVEL.	NEVER ATTENDED SCHOOL = 0<br>PRIMARY SCHOOL = 1<br>SECONDARY SCHOOL = 2<br>HIGH SCHOOL = 3<br>UNIVERSITY/COLLEGE = 4<br>POSTGRADUATE = 5<br>DON'T KNOW/DON'T REMEMBER = 8<br>REFUSED/NO ANSWER = 9	
105	What is your main current occupation?	UNEMPLOYED = 0<br>STAY AT HOME WIFE/HUSBAND = 1<br>FARMER = 2<br>SMALL TRADE (SELF-BUSINESS) = 3<br>WORKER = 4<br>GOVERNMENT EMPLOYEE = 5<br>PRIVATE COMPANY/ORGANISATION EMPLOYEE = 6<br>RETIRED = 7<br>STUDENT = 8<br>REFUSED/NO ANSWER = 9	If retired ⇒ 105a/b, otherwise skip to 105d

<table>
<tr><td></td><td></td><td>105_1: OTHER (SPECIFY)..</td><td></td></tr>
<tr><td>105a</td><td>If you are retired, when did you retire?
‘Unknow’ date is marked as 99
‘Unknow’ month is marked as 99
‘Unknow’ year is marked as 9998
‘Refused/no answer’ is marked as 9999</td><td>YEAR OF RETIREMENT _____/_______/________</td><td></td></tr>
<tr><td>105b</td><td>If year unknown:
How old were you when your retired?</td><td><table><tr><td>AGE (YEARS)</td><td>DON’T KNOW</td><td>REFUSED/NO ANSWER</td></tr><tr><td></td><td>888</td><td>999</td></tr></table></td><td></td></tr>
<tr><td>105c</td><td>What was your main occupation prior to retirement?</td><td><table><tr><td>WORKER</td><td>1</td></tr><tr><td>ARMY</td><td>2</td></tr><tr><td>POLICE</td><td>3</td></tr><tr><td>GOVERNMENT EMPLOYEE</td><td>4</td></tr><tr><td>PRIVATE COMPANY/ORGANISATION EMPLOYEE</td><td>5</td></tr><tr><td>OTHER</td><td>6</td></tr><tr><td>REFUSED/NO ANSWER</td><td>9</td></tr></table>105c_a: IF OTHER, SPECIFY:___________________________</td><td></td></tr>
<tr><td>105d</td><td>What is your household monthly income?</td><td><table><tr><td>VND</td><td>DON’T KNOW /DON’T REMEMBER</td><td>KHÔNG TRẢ LỜI / TỪ CHỐI</td></tr><tr><td></td><td>8</td><td>9</td></tr></table></td><td></td></tr>
<tr><td>106</td><td>How would you yourself assess the economic situation of your household? (Read up options)</td><td><table><tr><td>POOR</td><td>1</td></tr><tr><td>NEAR POOR</td><td>2</td></tr><tr><td>MEDIUM</td><td>3</td></tr><tr><td>WEALTHY</td><td>4</td></tr><tr><td>DON'T KNOW</td><td>8</td></tr><tr><td>REFUSED/NO ANSWER</td><td>9</td></tr></table></td><td></td></tr>
<tr><td>107</td><td>How do local authorities assess the economic situation of your household? (Read up options)</td><td><table><tr><td>POOR</td><td>1</td></tr><tr><td>NEAR POOR</td><td>2</td></tr><tr><td>UNCATEGORIZED</td><td>3</td></tr><tr><td>DON'T KNOW</td><td>8</td></tr><tr><td>REFUSED/NO ANSWER</td><td>9</td></tr></table></td><td></td></tr>
<tr><td>108</td><td>Are you married? If yes, do you live together?</td><td><table><tr><td>SINGLE</td><td>0</td></tr><tr><td>CURRENTLY MARRIED AND LIVING TOGETHER</td><td>1</td></tr><tr><td>CURRENTLY MARRIED BUT LIVING APART</td><td>2</td></tr><tr><td>LIVING TOGETHER, BUT NOT MARRIED</td><td>3</td></tr><tr><td>DIVORCED / SEPARATED</td><td>4</td></tr><tr><td>WIDOWED</td><td>5</td></tr><tr><td>REFUSED/NO ANSWER</td><td>9</td></tr></table></td><td>IF WIDOWED ⇒ 108A OTHERWISE ⇒ 109</td></tr>
</table>

STUDY INFORMANT ID No: __________

SECTION 2
HEALTH AND USE OF HEALTH CARE SERVICES

I would now like to ask a few questions about your health and use of health services.

201	In general, how would you describe your overall physical health?<br><br>(Read up options)	EXCELLENT 1<br>GOOD 2<br>FAIR 3<br>POOR 4<br>VERY POOR 5<br>REFUSED/NO ANSWER 9	
202	In general, how would you describe your overall mental health?<br><br>(Read up options)	EXCELLENT 1<br>GOOD 2<br>FAIR 3<br>POOR 4<br>VERY POOR 5<br>REFUSED/NO ANSWER 9	
203	Have you ever been diagnosed with the following medical conditions (other than diabetes)?	see table below	
204	**When** did you get the diagnosis of diabetes?<br>Please specify day, month and year:<br>'Unknow' date is marked as 99<br>'Unknow' month is marked as 99	___/_____/_____ (DD/MM/YYYY)	IF DATE KNOWN ⇒ 205

Response options for 201 and 202:

EXCELLENT	1
GOOD	2
FAIR	3
POOR	4
VERY POOR	5
REFUSED/NO ANSWER	9

Conditions for 203:

		NO	YES	REFUSED/ NO ANSWER
203_1	HYPERTENSION	0	1	9
203_2	EPILEPSY	0	1	9
203_3	DEPRESSION (Please probe)	0	1	9
203_4	TUBERCULOSIS	0	1	9
203_5	LIVER AND KIDNEY DISEASE	0	1	9
203_6	BONE AND JOINT PROBLEM	0	1	9
203_7	Cardio-vascular disease	0	1	9
203_8	ANY OTHER CHRONIC DISEASE	0	1	9

203_8a: IF OTHER SPECIFY: ________________________________
____

	‘Unknow’ year is marked as 9998<br>‘Refused/no answer’ is marked as 9999		
204a	How old were you when were diagnosed with diabetes?	AGE (YEARS) / DON’T KNOW: 888 / REFUSED/NO ANSWER: 999	
204b	**IF date unknow, ask:**	MORE THAN 8 YEARS AGO: 1<br>3-8 YEARS AGO: 2<br>1-3 YEARS AGO: 3<br>LESS THAN 1 YEAR AGO: 4<br>LESS THAN 1 MONTH AGO: 5<br>DON’T KNOW: 8<br>REFUSED/NO ANSWER: 9	
208	What type of medication do you take for your diabetes?	NO MEDICATION: 0<br>ORAL MEDICATION: 1<br>INSULIN/ INJECTIONS: 2<br>BOTH: 3<br>REFUSED: 9	
208a	Specify all types of medication prescribed in the patients ’patient-book’ AT LAST CHECK-UP:<br>1.____<br>2.____<br>3.____<br>4.____<br>5.____<br>6. ____<br>7. ____<br>8. ____		
208b	In the past 4 weeks, how often did you take the medications the doctor prescribed **for your diabetes**:<br>(Read up all options)	NO: 0<br>YES - ONCE OR TWICE PER MONTH: 1<br>YES - A FEW TIMES WEEKLY: 2<br>EVERY DAY: 3<br>DONT KNOW: 8<br>REFUSED NO ANSWER: 9	IF 3 move to 208d

STUDY INFORMANT ID No: __________

208c	Why didn't you take the medication the doctor prescribed for your diabetes? (MULTIPLE CHOICES)					
			NO	YES	DONT KNOW	REFUSED/ NO ANSWER
	208c_1	CONTROL BLOOD GLUCOSE LEVEL BY DIET	0	1	8	9
	208c_2	THINK THAT BLOOD GLUCOSE IS NOT HIGH	0	1	8	9
	208c_3	WORRY ABOUT NEGATIVE EFFECTS OF DIABETIC MEDICINE	0	1	8	9
	208c_4	OUT OF MEDICINE, NO TIME TO TAKE FROM HOSPITAL	0	1	8	9
	208c_5	OUT OF MEDICINE, NO MONEY TO BUY OR NO TIME TO BUY	0	1	8	9
	208c_6	OTHER	0	1	8	9
	208c_6a	IF OTHER, SPECIFY:____				

208d	SPECIFY MEDICATION YOU TAKE FOR YOUR DIABETES THAT ARE NOT PRESCRIBED BY THE DOCTOR?<br>1.________________________________<br>2.________________________________<br>3.________________________________<br>4.________________________________<br>5.________________________________<br>6. ________________________________<br>7. ________________________________<br>8. ________________________________
208e	SPECIFY MEDICATION YOU TAKE IN THE PAST 4 WEEK<br>(IN CASE, PATIENTS DON'T KEEP THE "PATIENTS-BOOK" AND THEY ALSO DON'T KNOW THE SOURCE OF THE MEDICATION THEY TOOK)<br>1.________________________________<br>2.________________________________<br>3.________________________________<br>4.________________________________<br>5.________________________________<br>6. ________________________________<br>7. ________________________________<br>8. ________________________________
208f	In the past 4 weeks, have you taken any of the following herbs **for your diabetes**:<br>(Read up all options)<br>FOR EACH **"YES" PROBE:**<br>How often? Once or twice, a few times weekly or daily?

	VIETNAMESE HERB/CHINESE HERB		NO	YES - ONCE OR TWICE	YES - A FEW TIMES WEEKLY	EVERY DAY	DONT KNOW	REFUSED NO ANSWER
	208f_1	VIETNAMESE HERBS (LEAF : GYMNEMA SYLVESTRE, GUAVA, MANGO…)	0	1	2	3	8	9

	208f_2	CHINESE HERBS	0	1	2	3	8	9	
	208f_3	FUNCTIONAL FOODS	0	1	2	3	8	9	
	208f_3a	If Yes, (1,2 or 3) for 208e_3, please specifiy:	______________________________________						
209	In the past 4 weeks, have you taken any other medications?:<br>(Read up all options)<br>FOR EACH **"YES" PROBE:**<br>How often? Once or twice, a few times weekly or daily?								
	**OTHER MEDICINES**<br>The same medication can be taken for different conditions		NO	YES - ONCE OR TWICE	YES - A FEW TIMES WEEKLY	EVERY DAY	DONT KNOW	REFUSED NO ANSWER	
	209_1	FOR HYPERTENTION __________	0	1	2	3	8	9	
	209_2	FOR EYES	0	1	2	3	8	9	
	209_3	FOR KIDNEY/LIVER	0	1	2	3	8	9	
	209_4	FOR NERVE	0	1	2	3	8	9	
	209_5	FOR OTHER DISEASE	0	1	2	3	8	9	

212	DO YOU USE MEDICINE IN HEALTH INSURANCE OR BUY YOURSELF? (Multiple options allowed)						
			NO	YES	DONT KNOW	REFUSED/NO ANSWER	If ONESELF -> 212c
	212_1	HEALTH INSURANCE	0	1	8	9	
	212_2	ONESELF	0	1	8	9	

212a	If you only use medicine in health insurance, do you have to spend money for diabetes medication?	No: 0<br>Yes: 1	If No, ->214
212b	If you have to spend money for diabetic medicine in health insurance, how much do you spend on medicaion per month?	VND: ___ / DON'T/DON'T REMEMBER: 8 / REFUSED/NO ANSWER: 9	
212c	If you have to buy medication by yourself. How much do you spend on diabetes medication per month?	VND: ___ / DON'T/DON'T REMEMBER: 8 / REFUSED/NO ANSWER: 9	
213	Why do you use diabetes medicine NOT COVERED (PAID) BY the health insurance?		

STUDY INFORMANT ID No: __________

			NO	YES	DONT KNOW	REFUSED/NO ANSWER	
	213_1	DON'T TRUST THE QUALITY OF INSURANCE MEDICINE	0	1	8	9	
	213_2	THINK THE INSURANCE MEDICATION IS LESS EFFECTIVE	0	1	8	9	
	213_3	TO PICK UP THE INSURANCE MEDICINE TAKE TOO MUCH TIME	0	1	8	9	
	213_4	INSURANCE MEDICINE HAVE MORE SIDE EFFECTS	0	1	8	9	
	213_5	OTHERS ENCOURAGE ME TO TAKE IT	0	1	8	9	
	213_6	GIVEN BY OTHER PEOPLE	0	1	8	9	
	213_7	OTHER	0	1	8	9	
		213_7a: IF OTHER, SPECIFY:______________________________					

219	I now have some questions about your feelings and experiences with living with diabetes. You can answer on a scale from 1 to 5 where 1 is not a problem and 5 is a serious problem<br>When answering the questions, please think about how your life has been during **the past four weeks:**								
			NOT A PROBLEM	MINOR PROBLEM	MODERATE PROBLEM	SOMEWHAT SERIOUS PROBLEM	SERIOUS PROBLEM	DON'T KNOW	
			1	2	3	4	5	8	
	219_1	Feeling scared when you think about living with diabetes	1	2	3	4	6	8	
	219_2	Feeling depressed when you think about living with diabetes	1	2	3	4	5	8	

	219_3	Worrying about the future and the possibility of serious compiications	1	2	3	4	5	8	
	219_4	Feeling that diabetes is taking up too much of your mental and physical energy every day	1	2	3	4	5	8	
	219_5	Coping with complications	1	2	3	4	5	8	

STUDY INFORMANT ID No: __________

Mã cá nhân......................................

Dự án "Hỗ trợ xã hội dành cho người bị bệnh tiểu đường"

MẪU PHỎNG VẤN SỐ 1

TÊN BỆNH NHÂN______________________

THÔN ____________________

XÃ ______________________

HUYỆN ____________________

SỐ CHỨNG MINH THƯ (NẾU CÓ)_____________________

NGÀY PHỎNG VẤN: ngày [ ][ ] tháng [ ][ ] năm [ ][ ][ ][ ]

000. THỜI GIAN BẮT ĐẦU PHỎNG VẤN	Giờ [ ][ ] (24 h)<br>Phút [ ][ ]
03. Tên điều tra viên<br><br>04. Tên giám sát viên	

STUDY INFORMANT ID No: __________

<table>
<tr><td colspan="4">PHẦN 1
THÔNG TIN VỀ NGƯỜI TRẢ LỜI VÀ GIA ĐÌNH</td></tr>
<tr><td colspan="2">NỘI DUNG CÂU HỎI</td><td>CÁC MÃ TRẢ LỜI</td><td>GHI CHÚ</td></tr>
<tr><td colspan="3">Nếu ông/bà không phiền, tôi xin được hỏi một số câu hỏi về cá nhân và gia đình ông/bà</td><td></td></tr>
<tr><td>101</td><td>Giới tính người trả lời</td><td>NAM: 0
Nữ: 1</td><td></td></tr>
<tr><td>102</td><td>Ông/bà sinh năm nào?
(NGÀY/THÁNG/NĂM)
Không nhớ ngày: ghi 99
Không nhớ tháng: ghi 99
Không nhớ năm: ghi 9998
Từ chối/Không trả lời: ghi 9999</td><td>NĂM SINH ___/_____/_____ (DƯƠNG LỊCH)
NĂM SINH ___/_____/_____ (ÂM LỊCH)</td><td>Nếu nhớ ngày, tháng ➔ Chuyển 103</td></tr>
<tr><td>102a</td><td>Nếu không nhớ ngày tháng năm sinh, Ông/bà năm nay bao nhiêu tuổi</td><td>TUỔI(NĂM):
KHÔNG BIẾT/KHÔNG NHỚ: 888
KHÔNG TRẢ LỜI / TỪ CHỐI: 999</td><td></td></tr>
<tr><td>103</td><td>Ông/bà lớn lên ở đâu?
GỢI Ý: từ nhỏ đến năm 18 tuổi ông bà sống ở đâu lâu nhất?</td><td>Ở XÃ NÀY: 1
Ở XÃ KHÁC NHƯNG CÙNG HUYỆN: 2
Ở HUYỆN KHÁC: 3
Ở TỈNH HOẶC THÀNH PHỐ KHÁC: 4
KHÔNG BIẾT/KHÔNG NHỚ: 8
KHÔNG TRẢ LỜI / TỪ CHỐI: 9</td><td></td></tr>
<tr><td>104</td><td>Trình độ học vấn của ông/bà?
CHỌN TRÌNH ĐỘ CAO NHẤT</td><td>CHƯA BAO GIỜ ĐI HỌC: 0
TIỂU HỌC: 1
TRUNG HỌC CƠ SỞ: 2
TRUNG HỌC PHỔ THÔNG: 3
ĐẠI HỌC/CAO ĐẲNG/TRUNG CẤP: 4
SAU ĐẠI HỌC: 5
KHÔNG BIẾT/KHÔNG NHỚ: 8
KHÔNG TRẢ LỜI / TỪ CHỐI: 9</td><td></td></tr>
<tr><td>105</td><td>Nghề nghiệp chính của ông/bà hiện nay?</td><td>THẤT NGHIỆP: 0
Ở NHÀ/NỘI TRỢ: 1
NÔNG DÂN: 2
BUÔN BÁN NHỎ: 3
CÔNG NHÂN: 4
LÀM TRONG CƠ QUAN NHÀ NƯỚC: 5
LÀM CHO CÔNG TY HOẶC TỔ CHỨC TƯ NHÂN: 6
NGHỈ HƯU: 7
SINH VIÊN: 8
KHÔNG TRẢ LỜI / TỪ CHỐI: 9
105_1: NGHỀ KHÁC (GHI RÕ): ________________________</td><td>Nếu đã nghỉ hưu, ⇒ chuyển câu 105a/b, nếu không, chuyển đến câu 105d</td></tr>
<tr><td>105a</td><td>Nếu đã nghỉ hưu, ông/bà nghỉ hưu khi nào?
Không nhớ ngày: ghi 99
Không nhớ tháng: ghi 99
Không nhớ năm: ghi 9998
Không/Từ chối trả lời: ghi 9999</td><td>NĂM NGHỈ HƯU _____/_______/________</td><td></td></tr>
</table>

105b	Nếu ông/bà không nhớ năm nghỉ hưu, Thì ông bà nghỉ hưu lúc bao nhiêu tuổi	TUỔI (NĂM) | KHÔNG BIẾT | KHÔNG TRẢ LỜI / TỪ CHỐI	| 888 | 999	
105c	Nghề chính của ông/bà trước khi nghỉ hưu?	CÔNG NHÂN	1	
		BỘ ĐỘI	2	
		CÔNG AN	3	
		LÀM TRONG CƠ QUAN NHÀ NƯỚC	4	
		LÀM CHO CÔNG TY HOẶC TỔ CHỨC TƯ NHÂN	5	
		KHÁC	6	
		KHÔNG TRẢ LỜI / TỪ CHỐI	9	
		105c_a: NGHỀ KHÁC (GHI RÕ):______________________________		
105d	HIện tại, Trung bình 1 gia đình ông/bà tháng thu nhập bao nhiêu? (Trung bình cả hộ gia đình)	VNĐ | KHÔNG BIẾT/ KHÔNG NHỚ | KHÔNG TRẢ LỜI / TỪ CHỐI	| 8 | 9	
106	Theo ông/bà, tình trạng kinh tế của gia đình ông/bà thuộc loại nào sau đây? (Người phỏng vấn đọc các câu trả lời)	NGHÈO	1	
		CẬN NGHÈO	2	
		TRUNG BÌNH	3	
		KHÁ GIẢ	4	
		KHÔNG BIẾT	8	
		KHÔNG TRẢ LỜI / TỪ CHỐI	9	
107	Tình trạng kinh tế nhà ông/bà được nhà nước xếp loại như thế nào? (Người phỏng vấn đọc các câu trả lời)	HỘ NGHÈO	1	
		HỘ CẬN NGHÈO	2	
		KHÔNG XẾP LOẠI	3	
		KHÔNG BIẾT	8	
		KHÔNG TRẢ LỜI / TỪ CHỐI	9	
108	Ông/bà có kết hôn không? Nếu có, ông/bà có sống cùng nhau không?	ĐỘC THÂN	0	NẾU LÀ GÓA ⇒ CHUYỂN CÂU 108A Nếu không Chuyển ⇒ 109
		ĐANG KẾT HÔN VÀ SỐNG CÙNG NHAU	1	
		ĐANG KẾT HÔN NHƯNG SỐNG XA NHAU	2	
		SỐNG NHƯ VỢ CHỒNG NHƯNG KHÔNG KẾT HÔN	3	
		LY THÂN/LY DỊ	4	
		GOÁ	5	
		KHÔNG TRẢ LỜI / TỪ CHỐI	9	

	**PHẦN 2**<br>**SỨC KHỎE VÀ VIỆC SỬ DỤNG CÁC DỊCH VỤ Y TẾ**
	Tôi muốn hỏi ông/bà một số câu hỏi về sức khỏe và việc sử dụng dịch vụ y tế của ông/bà.

201 Nhìn chung, ông/bà cảm nhận sức khỏe của mình là?

(Đọc rõ các lựa chọn)

RẤT TỐT	1
TỐT	2
TẠM ĐƯỢC	3
YẾU	4
RẤT YẾU	5
KHÔNG TRẢ LỜI / TỪ CHỐI	9

202 Nhìn chung, ông/bà cảm nhận tình trạng tinh thần của mình là?

(Đọc rõ các lựa chọn)

RẤT TỐT	1
TỐT	2
TẠM ĐƯỢC	3
KHÔNG ỔN	4
RẤT KHÔNG ỔN	5
KHÔNG BIẾT	8
KHÔNG TRẢ LỜI / TỪ CHỐI	9

203 Ngoài bệnh tiểu đường, ông/bà có từng được chẩn đoán các bệnh nào sau đây không?

		KHÔNG	CÓ	KHÔNG TRẢ LỜI / TỪ CHỐI
203_1	CAO HUYẾT ÁP	0	1	9
203_2	ĐỘNG KINH	0	1	9
203_3	TRẦM CẢM	0	1	9
203_4	LAO	0	1	9
203_5	GAN/THẬN	0	1	9
203_6	BỆNH XƯƠNG KHỚP	0	1	9
203_7	CÁC BỆNH VỀ TIM	0	1	9
203_8	BỆNH MẠN TÍNH KHÁC	0	1	9

203_8a BỆNH KHÁC (GHI RÕ): ________________________________

204 Ông/bà được chẩn đoán bị tiểu đường khi nào?
Ghi rõ ngày, tháng, năm

Không nhớ ngày: ghi 99
Không nhớ tháng: ghi 99
Không nhớ năm: ghi 9998
Không/Từ chối trả lời: ghi 9999

_____/_______/_________ (Ngày/tháng/năm)

Nếu nhớ ngày thì chuyển → 205

204a Khi được chẩn đoán tiểu đường Ông/bà bao nhiêu tuổi?

TUỔI (NĂM)	KHÔNG BIẾT/ KHÔNG NHỚ	KHÔNG TRẢ LỜI / TỪ CHỐI
	888	999

204b Nếu không nhớ chính xác được ngày bị, ông /bà ước chừng khoảng thời gian đó là từ khi nào?

HƠN 8 NĂM TRƯỚC	1
3-8 NĂM TRƯỚC	2
1-3 NĂM TRƯỚC	3
GẦN 1 NĂM TRƯỚC	4
GẦN 1 THÁNG TRƯỚC	5
KHÔNG BIẾT	8
KHÔNG TRẢ LỜI / TỪ CHỐI	9

208 **Ông/bà dùng các thuốc tiểu đường nào sau đây?**

KHÔNG DÙNG THUỐC	1
CHỈ THUỐC UỐNG	2

	CHỈ INSULIN	3		
	CẢ THUỐC TIÊM VÀ THUỐC UỒNG	4		
	KHÔNG TRẢ LỜI	9		

208a ĐIỀU TRA VIÊN VIẾT LẠI TOÀN BỘ CÁC THUỐC ĐƯỢC KÊ TRONG SỔ KHÁM BỆNH (GẦN NHẤT)

1____
2____
3.____
4____
5____
6.____
7____
8____

208b **Trong 4 tuần qua, Ông/bà có thường xuyên dùng các thuốc được bác sĩ kê đơn không?**

Nếu 3, Chuyển 208 d

Không	0
Có, 1,2 lần 1 tháng	1
Có, vài lần /tuần	2
Hàng ngày	3
Không biết	8
Từ chối trả lời	9

208c Nếu không uống thuốc thường xuyên, Lý do tại sao ông/bà không dùng thuốc theo đơn của bác sĩ (nhiều lựa chọn)

		KHÔNG	CÓ	KHÔNG BIẾT	KHÔNG TRẢ LỜI / TỪ CHỐI
208c_1	CÓ THỂ TỰ KIỂM SOÁT ĐƯỜNG BẰNG ĂN UỐNG	0	1	8	9
208c_2	THẤY ĐƯỜNG MÁU CHƯA CAO	0	1	8	9
208c_3	SỢ TÁC DỤNG PHỤ CỦA THUỐC	0	1	8	9
208c_4	MỚI HẾT THUỐC CHƯA ĐI LẤY THUỐC BẢO HIỂM ĐƯỢC	0	1	8	9
208c_5	HẾT THUỐC, CHƯA MUA (KHÔNG DÙNG THUỐC BẢO HIỂM)	0	1	8	9
208c_6	KHÁC	0	1	8	9
208c_6 a	GHI RÕ:____				

208d ĐIỀU TRA VIÊN VIẾT LẠI TOÀN BỘ CÁC THUỐC TIỂU ĐƯỜNG BỆNH NHÂN **ĐANG DÙNG MÀ KHÔNG ĐƯỢC KÊ** TRONG SỔ KHÁM BỆNH (GẦN NHẤT)

1____
2____
3.____
4____
5____

	6.___<br>7___<br>8___	
208e	NẾU BỆNH NHÂN KHÔNG CÓ SỔ KHÁM BỆNH (NGƯỜI KHÁC CẦM ĐỂ LẤY THUỐC GIÚP) VÀ CŨNG KHÔNG BIẾT THUỐC ĐANG UỐNG LÀ THUỐC BẢO HIỂM HAY MUA THÊM. THÌ GHI LẠI TOÀN BỘ NHỮNG THUỐC BỆNH NHÂN ĐANG UỐNG<br><br>1___<br>2___<br>3.___<br>4___<br>5___<br>6.___<br>7___	

208f		**Trong 4 tuần qua, Ông/bà có dùng các thuốc sau đây không?**<br>VỚI MỖI CÂU TRẢ LỜI **"CÓ" GỢI Ý:** Mức độ thường xuyên? Một, hai hoặc nhiều lần mỗi tuần?<br>(Điều tra viên đọc các lựa chọn)							
	**THUỐC BẮC/THUỐC NAM**		KHÔNG	CÓ, 1LẦN/TUẦN	CÓ, VÀI LẦN/TUẦN	CÓ, UỐNG HÀNG NGÀY	KHÔNG BIẾT	KHÔNG TRẢ LỜI / TỪ CHỐI	
	208f_1	THUỐC NAM (CÁC LOẠI LÁ CÂY (THÌA CANH, ỔI, XOÀI…)	0	1	2	3	8	9	
	208f_2	THUỐC BẮC	0	1	2	3	8	9	
	208f_3	THỰC PHẨM CHỨC NĂNG	0	1	2	3	8	9	
	208f_3 a	Nếu có dùng thực phẩm chức năng, GHI RÕ TÊN							
209		**Trong 4 tuần qua, Ông/bà có dùng các thuốc sau đây không?**<br>VỚI MỖI CÂU TRẢ LỜI **"CÓ" GỢI Ý:** Mức độ thường xuyên? Một, hai hoặc nhiều lần mỗi tuần?<br>(Điều tra viên đọc các lựa chọn)							
	**CÁC THUỐC KHÁC**		KHÔNG	CÓ, 1LẦN/TUẦN	CÓ, VÀI LẦN/TUẦN	CÓ, UỐNG HÀNG NGÀY	KHÔNG BIẾT	KHÔNG TRẢ LỜI / TỪ CHỐI	
	209_1	THUỐC TĂNG HUYẾT ÁP	0	1	2	3	8	9	
	209_2	THUỐC BỔ MẮT	0	1	2	3	8	9	
	209_3	THUỐC BỔ GAN/THẬN	0	1	2	3	8	9	
	209_4	THUỐC BỔ THẦN KINH	0	1	2	3	8	9	
	209_5	THUỐC KHÁC	0	1	2	3	8	9	
	209_5a CÁC THUỐC KHÁC, GHI RÕ___								

212	Ông/bà dùng thuốc tiểu đường trong bảo hiểm y tế hay tự mua thuốc? (Nhiều lựa chọn)		KHÔNG	CÓ	KHÔNG BIẾT	KHÔNG TRẢ LỜI / TỪ CHỐI	**Nếu TỰ MUA, CHUYỂN -> 212c**
	212_1	THUỐC BẢO HIỂM	0	1	8	9	
	212_2	TỰ MUA	0	1	8	9	

212a	Nếu chỉ dùng thuốc bảo hiểm, ông bà có phải trả thêm tiền thuốc không?	Không: 0<br>Có: 1	**Nếu KHÔNG, chuyển 214**
212b	Nếu phải trả tiền thuốc bảo hiểm, một tháng ông/bà phải trả bao nhiêu?	VND: ___ / KHÔNG BIẾT /KHÔNG NHỚ: 8 / KHÔNG TRẢ LỜI / TỪ CHỐI: 9	
212c	Nếu tự mua thuốc, tiền thuốc tiểu đường là bao nhiêu?	VND: ___ / KHÔNG BIẾT /KHÔNG NHỚ: 8 / KHÔNG TRẢ LỜI / TỪ CHỐI: 9	

213 Tại sao ông bà phải mua thuốc tiểu đường bên ngoài bảo hiểm y tế?

		KHÔNG	CÓ	KHÔNG BIẾT	KHÔNG TRẢ LỜI / TỪ CHỐI
213_1	Không tin tưởng chất lượng thuốc bảo hiểm	0	1	8	9
213_2	Thuốc bảo hiểm nhẹ quá	0	1	8	9
		KHÔNG	CÓ	KHÔNG BIẾT	KHÔNG TRẢ LỜI / TỪ CHỐI
213_3	Đi lấy thuốc bảo hiểm phải đi xa, không đi lấy được	0	1	8	9
213_4	Thuốc bảo hiểm nhiều tác dụng phụ	0	1	8	9
213_5	Người khác khuyên dùng	0	1	8	9
213_6	Con/cháu mua cho nên uống	0	1	8	9
213_7	KHÁC	0	1	8	9
	213_7a: GHI RÕ:___				

219 Bây giờ tôi sẽ hỏi những câu hỏi về cảm nhận cũng như những trải nghiệm của ông/bà khi sống cùng bệnh tiểu đường.
Khi trả lời câu hỏi, ông/bà hãy nghĩ đến cuộc sống của mình trong vòng **4 tuần qua:**
Ông bà hãy trả lời theo thang đo từ 1 đến 5,
với 1: điều đó không có vấn đề gì và 5 đó là vấn đề rất nghiêm trọng

		Không vấn đề gi	Vấn đề nhỏ	Vấn đề khá nghiêm trọng	Vấn đề nghiêm trọng	Vấn đề rất nghiêm trọng	KHÔNG BIẾT	KHÔNG TRẢ LỜI / TỪ CHỐI
		1	2	3	4	5	8	9
219_1	Tôi cảm thấy sợ hãi khi nghĩ đến việc phải sống chung với bệnh tiểu đường.	1	2	3	4	5	8	9
219_2	Tôi cảm thấy chán nản khi nghĩ đến việc phải sống	1	2	3	4	5	8	9

		chung với bệnh tiểu đường.							
	219_3	Tôi lo lắng về tương lai và nguy cơ biến chứng nghiêm trọng	1	2	3	4	5	8	9
	219_4	Tôi thấy bệnh tiểu đường gây tổn hại quá nhiều về thể chất và tinh thần mỗi ngày…	1	2	3	4	5	8	9
	219_5	Tôi thấy khó khăn khi đối mặt với biến chứng của bệnh tiểu đường	1	2	3	4	5	8	9
